# Development and validation of a questionnaire assessing the quality of life impact of Colour Blindness (CBQoL)

**DOI:** 10.1186/s12886-017-0579-z

**Published:** 2017-10-02

**Authors:** John A. Barry, Susan Mollan, Michael A. Burdon, Michelle Jenkins, Alastair K. Denniston

**Affiliations:** 1 Clinical, Educational and Health Psychology, University College London, London, WC1E 6BT UK; 2 Department of Ophthalmology, Queen Elizabeth Hospital Birmingham, Birmingham, B15 2WB UK; 3 Department of Psychology, Open University, Milton Keynes, UK; 4 Academic Unit of Ophthalmology, Institute of Inflammation & Ageing, University of Birmingham, Birmingham, B15 2TT UK

**Keywords:** Colour vision deficiency, Colour blind, Quality of life, Psychology, Questionnaire, Factor analysis, Principal components analysis

## Abstract

**Background:**

Congenital colour vision deficiency (CVD), commonly called ‘colour blindness’, affects around 8% of men and 0.4% of women. Although many aspects of health (e.g. change in colour of urine) and healthcare (e.g. coloured medication, colour-coded diagnostic tests), and modern life depend upon colour coding (e.g. graphs, maps, signals), the impact of colour blindness on everyday life is not generally considered a topic of importance. This study is the first to create and validate a questionnaire measuring the quality of life (QoL) impact of being colour blind.

**Methods:**

This study consisted of two phases. Firstly, the questionnaire design and development phase was led by an expert panel and piloted on a focus group. Secondly, an online sample of 128 men and 291 women filled in the questionnaire, and the psychometric properties of the questionnaire were analysed using principal components analysis (PCA). The scores of colour blind (CB) participants and normal-sighted controls, controlling for age and sex, were compared using matched t-tests.

**Results:**

The PCA resulted in a questionnaire with three domains (or subscales): QoL for Health & Lifestyle, QoL for Work, and QoL for Emotions. Controlling for age, there was a significantly greater negative impact on QoL for CB people than normal-sighted controls in regards to confusion over colour in various aspects of their health (*p* = 5 × 10^−7^), work (*p* = 1.3 × 10^−7^), and emotional life (*p* = 6 × 10^−5^).

**Conclusion:**

Colour blindness can significantly impact quality of life for health, emotions, and especially careers. The tool developed here could be useful in future clinical studies to measure changes in CBQoL in response to therapy in conditions where colour vision is affected. We also discuss ways in which everyday problems related to colour vision might be reduced, for example, workplaces could avoid colour coding where a non-colour alternative is possible.

## Background

Colour blindness (CB) (also known as colour vision deficiency, CVD) is a common condition, with around 8% of men and 0.4% of women being affected from birth (i.e. congenital CB) [1]. Many aspects of modern life increasingly require accurate colour vision [2], from colour-coded maps and graphs, to electronic wiring & components, to signalling & communications, and even to reading text set in a coloured background. Despite the prevalence of this condition, and the many restrictions that being colour blind puts on various aspects of life, there has been surprisingly little research on the quality of life (QoL) impact of congenital colour blindness.

In their review of literature on the impact of colour blindness on everyday functioning, Chan et al. describe effects across the lifespan [3]. In ‘play age’, children will experience learning difficulties (e.g. coloured chalk on a blackboard), impaired social play (e.g. colours of team clothes in sports). Children may also experience behavioural issues (e.g. embarrasment and social withdrawl, or being a fussy eater due to unappetising appearance of colour of foods such as vegetables). Colour blind schoolchildren may fall behind in subjects where colours are a necessary part of learning, and colour blindness may be treated as “something of a joke” (Sullivan 2011, p.21) [4].

The problems experienced in younger life may continue into adulthood, especially for those who have not received a diagnosis. Career choices for young adults may be restricted, for example, the armed forces rely on colours for signaling, and colour is important in the sciences [5, 6]. Such problems extend into middle and late adulthood, with restrictions at work, problems with driving safely, and problems taking coloured medication correctly [2]. Tagarelli et al. found that 66–90% of colour blind people have problems with everyday tasks, such as reading charts or knowing when meat is cooked properly [7].

Although validated questionnaires exist that measure QoL aspects of visual functioning, these are designed to measure the general impact of ocular pathology rather than colour vision problems. Moreover, they are very limited in their ability to assess the impact of colour vision deficiency on QoL, for example, the popular VFQ-25 - and even the larger NEI-VFQ - contain only one item (choosing / matching clothing) to measure colour vision problems [8].

The aim of this study was to develop and validate a QoL measure of colour blindness, and determine whether colour blindness has a significant impact on quality of life compared to people with normal colour vision.

## Method

### Design

This study was an online survey analysed using standard questionnaire validation methods, such as principal components analysis. People with CB were involved in the design and implementation of this study.

This first part of this paper describes the development of the questionnaire, and the second part describes the validation of the questionnaire. In the second part, the scores of people with CB are compared with the scores of people with normal colour vision.

### Setting

The setting of this study was online.

### Participants

Participants were recruited from various sources, mostly online (details in the section *Initial Validation of Final CB Questionnaire,* below). They were categorised as being CB or not, as described in the *Materials* section below. The authors use the common term ‘colour blindness’ (CB) for the sake of clarity, but recognize that participants in fact had varying degrees of colour vision deficiency (CVD). Characteristics of the sample are shown in Table 1 below.

**Table 1:** Description of the colour vision and social background of the participants by sex (128 men and 291 women)

			Colour vision category			*χ* ^2^
			CB (*N* = 65)	Borderline (*N* = 22)	Normal vision (*N* = 332)	
Sex	Male		57 (88%)	6 (27%)	65 (20%)	118.984^a^
	Female		8 (12%)	16 (73%)	267 (80%)	
	SEC	Managerial	38 (65%)	10 (56%)	183 (68%)	4.328^b^
		Intermediate	5 (9%)	0 (0%)	24 (9%)	
		Manual	15 (26%)	8 (44%)	63 (23%)	

*CB* colour blind (Ishihara score 0–9), Borderline normal vision (Ishihara score 10–12), Normal vision (Ishihara score 13–15). *SEC* Socioeconomic class. Note 17% (73 of 419) of SEC responses were missing or uncategorisable, thus totals do not add up to Ns for each group as a whole

^a^ Sig value: *p* = 1.5 × 10^−26^

^b^ Correction with Fisher’s exact test, as three cells had expected frequencies of less than 5

Ethical approval was granted by the University College London Research Ethics Committee (Project ID: 4075/004). Informed consent was given by the participants before filling in the questionnaire.

#### Phase 1: development of CB questionnaires

Items for the questionnaire were developed through (a) a literature search, (b) an online lay focus group consisting of six individuals with CB and two normally-sighted people, recruited via *Psychology on The Net* and snowball sampling, and (c) an expert focus group consisting of three consultant ophthalmologists (SM, MAB, AKD), and a psychologist (JB) who has published several papers on questionnaire development in health and psychological conditions. JB has congenital deuteranopia (‘red-green’ CB). At these meetings, discussion focused on the types of experiences that were important to people with CB. Three main themes emerged: the career impact of being CB, the emotions associated with being CB, and the lifestyle impact of being CB. These various experiences were listed and then phrased as questions, with appropriate Likert scales added. Through this process, the 36 items that formed the basis of the questionnaire  were derived.

Six-point Likert scales were used, with lower scores indicating worse QoL. Responses were on a 6-point Likert scale from 1 = *A severe problem*, to 6 = *No problem*, with an option for ‘not applicable’. In this development phase, the questionnaire was administered to six CB people and two normal vision (NV) controls. The survey was completed online. All suggestions for revisions to the survey were recorded and changes to the questionnaires were made based on these suggestions. For example, an item was added regarding judging by colour whether food is sufficiently cooked. Also, the background colour of the survey was changed so that the questionnaire was easier for CB people to use.

A principal components analysis (PCA) was conducted to examine the factor structure of the questionnaire. The PCA used Varimax rotation and Kaiser normalization. Missing data were deleted pairwise, so that where a participant gave some answers but had not completed the questionnaire, the responses they gave could be included in the analysis. Extraction and retention of factors was based on visual examination of the scree plot [9] and eigenvalues of >1.0 were retained [10]. The threshold for the Kaiser-Meyer-Olkin (KMO) Measure of Sampling Adequacy was 0.6 [11]. Cronbach’s *α* coefficient values were assessed [12] in order to measure the internal reliability of a questionnaire. The usual threshold for acceptability for Cronbach’s *α* is 0.7 [13]. A factor loading threshold of .60 was applied to enhance the strength of factors, so only items of this strength, or with loadings that could be rounded up to .60, were retained. For factor loadings of .6 to be significant, a minimum of 85 participants are required to allow sufficient statistical power [14].

#### Phase 2: initial validation

##### Initial validation of final CB questionnaire

For the initial validation of the questionnaire, an online survey invited individuals with CB and NV controls to fill in the questionnaire. Participants were recruited between Sept 2014 and Sept 2015 from relevant websites and social media sources, including *Colour Blind Awareness*, the *Men’s Health Forum*, *Psychology on The Net*, *Online Psychology Research* and Birmingham University’s *Medical School Newssheet.*

### Materials

#### Ishihara colour test

Colour blindness was assessed using the Ishihara Colour Test [15]. This is a set of up to 24 coloured plates [16] in which the visibility of numbers or letters will depend upon the colour vision of the viewer. The plates are scored by giving one point for each plate correctly identified. This study used 15 plates, the number required to identify colour deficiency. Those who scored up to 9 plates correctly were categorized as colour blind; those scoring 13 or more were considered to have normal colour vision. For the purposes of our study, those who scored between 10 and 12 were categorized as borderline colour blind.

#### Health-related Quality of Life (HRQoL)

To assess the degree to which CB might have an impact on health-related quality of life, the participants filled in the Short Form 36 (SF-36) [17]. This 36-item health questionnaire is widely used and assesses QoL for eight dimensions of health. The subscales are rated on Likert scales, with lower scores indicating worse health. The response format of the scales varies, for example, ‘excellent’ to ‘poor’ for one item, and ‘not at all’ to ‘all the time’ for another. For the purpose of the present study, only the mental health (SF-36 MH) subscale was used. The Cronbach’s alpha for this subscale is 0.926.

#### Positive state of mind

To assess the degree to which CB might impact mental health, The Positive Mindset Index (PMI) [18] was administered to participants. This scale consists of six items (happiness, confidence, being in control, emotional stability, motivation and optimism) on a 5-point Likert scale. This scale shows good internal reliability (Cronbach’s alpha = 0.926) and shows good concurrent validity with measures of mental health [18, 19].

### Characteristics of the sample

Table 1 shows the characteristics of the sample.

As expected, there were significantly more men than women with colour blindness (*χ* ^2^ = 118.98, df = 2, *p* = 1.5 × 10^−26^). Of the 65 CB participants, four (6%) reported that their CB was acquired rather than congenital. There was no significant difference in the socioeconomic background distributions in the three vision groups, with 56–68% coming from a professional background, 23–44% from a manual background, and 0–9% from an intermediate background.

The mean (SD) age of the CB group (44.8 ± 15.6 years) was significantly older than that of the NV group (30.1 ± 15.1 years) (*p* = 9 × 10^−11^). In order to reduce any effect of age on outcomes, matching of participants in each group by age was carried out.

Of the 56 CB participants aged 18 to 65 years old, it was possible to match 30 of them by age to the nearest year to one or more NV community controls. This age limit of 65 years was chosen both because it spans the average working age in the UK and avoids the general declining of vision which is common with older age. Where there was more than one person in a group of the same age, the mean of their scores was used. For example, for participants aged 30 years old, there was one CB and three NV controls, thus the sole CB score was compared to the mean of the three NV scores. In some cases there was more than one CB participant of the same age e.g. at age 45 the mean of four CB participants was paired with the mean of six controls. Using this process, 30 age-matched pairs were possible from 46 CB and 199 control participants. Because men and women typically have different rates and types of colour blindness, the groups were further subdivided by sex. Fewer age-matched pairings were possible within each sex: 11 pairings of men (16 CB and 18 controls) and five pairings of women (five CB and 20 controls).

### Initial validation analysis

As a first step in validating a newly developed questionnaire, the construct validity of the questionnaire can be tested by assessing differences in scores between groups who are known to be different in relevant ways. In the present study, the two groups were the CB and normal sighted controls, matched for age. The mean scores on the Ishihara test, CBQoL, PMI and SF-36 MH were compared using matched t-tests. In the QoL questionnaire subscales, a higher score indicated a better QoL. All statistical analyses were carried out using SPSS statistical software for Windows, Version 22 (Armonk, NY: IBM Corp). The significance threshold was set at .05, and all *P* values were 2-tailed.

A further step in validating new questionnaires is testing how much they are in agreement with existing validated questionnaires measuring similar constructs. This is known as concurrent validity, and acceptable concurrent validity is indicated by a Pearson’s correlation coefficient (*r*) of 0.5 or more [20]. The criterion by which the new questionnaire subscales were measured was the Ishihara Colour Blindness Test [15], which assesses colour vision acuity. Thus concurrent validity between the CBQoL questionnaire and the Ishihara was assessed in this way.

To assess concurrent validity with health and psychological aspects of QoL, two other questionnaires were used. Firstly, the QoL mental health subscale of the SF-36 [17], on which lower scores indicate worse mental health. Secondly, the Positive Mindset Index (PMI) [18] was used, on which higher scores represent a more positive state of mind.

## Results

### Development of final CBQoL questionnaire

The mean (SD) time taken to complete the survey was 15.26 (±10.28) minutes. The CBQoL consisted of 36 items, identified as being issues relevant to being colour blind. The stimulus question was: *“Some people have had difficulties related to colours in their everyday life, regardless of whether they have a diagnosis of colour blindness or not. Please answer the following questions, which are about how much seeing colours may have been a problem for you. Please answer the questions whether you have a diagnosis of colour blindness or not. For each of the questions below, please state how much any of the following situations have ever been a problem for you because of difficulty in seeing colours properly”.*

Responses were on a 6-point Likert scale from 1 = *A severe problem*, to 6 = *No problem*, with an option for ‘not applicable’. The scores on the subscales (Table 2) were converted to means (Table 3) with a maximum score of 6 and minimum of 1. The 36 items relating to problems caused by colour confusion were: career limitations, avoiding aspects of work, underachievement, problems with coloured charts, coloured text, buying clothes, driving, reading maps, avoiding discussions involving colours, caused problems in family, social activities, socializing, ripeness of fruit, food cooked fully, choosing groceries, coloured medication, sunburn, mole on skin, urine, blood in faeces, avoiding activities, felt let others down, embarrassment, depression, anxiety, unconfident, feeling different, self-esteem, annoyed, worried, problems with sports, problems in dim lighting, pain, concentration, confusion, and lethargy. For the purposes of this initial research into CVD, there were also free text questions to allow participants to state other emotional and physical symptoms not in the list; the free text responses did not add a great deal to the statistical information and are not discussed below, and are not part of the CBQoL.

**Table 2:** Principal components analysis of CBQoL items

	Component		
Items	Health & Lifestyle	Emotions	Work
Not noticing change in colour of skin due to sunburn	.810		
Difficulty choosing groceries due to colour	.766		
Not noticing change in colour of mole on skin	.763		
Can’t tell when food is cooked due to colour	.755		
Difficulty choosing or buying clothes	.749		
Being confused about colour of pills or other medication due to colour-coding	.733		
Not noticing blood in stools (faeces)	.718		
Difficulty knowing when fruit is ripe due to colour	.711		
Difficulty reading maps (e.g. London Underground map)	.707		
Not noticing a change in colour of urine	.679		
Problems playing sports (e.g. colours of team clothing, colours of snooker balls etc)	.630		
Feeling anxious because of issues caused by problems seeing colours		.880	
Feeling depressed because of issues caused by problems seeing colours		.846	
Feeling unconfident because of issues caused by problems seeing colours		.820	
Feeling embarrassed or humiliated because of CB issues		.816	
Feeling low self esteem because of issues caused by problems seeing colours		.778	
Feeling anxious because you might not realise when you can’t see a colour properly		.767	
Feeling different to other people because of issues caused by problems seeing colours		.720	
Felt that had let down self or others due to problems seeing colours		.692	
Avoiding conversations where colours are discussed		.627	
Being limited in choice of work or career			.754
Difficulty performing work or other activities (e.g. charts)			.658
Accomplishing less than would like at work or in career			.621

**Table 3:** Mean (SD) scores for the male and female age-matched participants separately.

	Age- and Gender-Matched				Age- and Gender-Matched				Age-Matched			
	Male				Female				All participants			
	(*N* = 11 pairings)				(*N* = 5 pairings)				(*N* = 30 pairings)			
	CB	Normal	*t*	*p* value	CB	Normal	*t*	*p* value	CB	Normal	*t*	*p* value
Ishihara	3.0 (1.3)	14.5 (0.7)	−32.2	1.9 × 10^−10^	4.2 (2.7)	14.9 (0.2)	−9.1	.001	3.4 (2.0)	14.6 (0.5)	−29.6	2.5 × 10^−23^
CBQoL Emotion	4.1 (1.9)	5.7 (0.5)	−2.8	.018	4.7 (1.6)	6.0 (0.0)	−1.9	(ns)	4.4 (1.5)	5.9 (0.2)	−5.5	6 × 10^−5^
CBQoL Health	3.8 (1.6)	5.7 (0.6)	−4.2	.002	4.1 (2.1)	6.0 (0.1)	−1.8	(ns)	4.1 (1.4)	5.8 (0.2)	−6.4	5 × 10^−7^
CBQoL Work	3.8 (1.8)	5.6 (0.7)	−4.5	.001	4.5 (1.6)	6.0 (0.2)	−1.9	(ns)	3.6 (1.6)	5.9 (0.2)	−7.8	1.3 × 10^−7^
Positive Mindset	3.8 (0.8)	3.8 (0.5)	0.2	(ns)	3.0 (0.8)	3.6 (0.2)	−1.8	(ns)	3.5 (0.7)	3.7 (0.3)	−1.4	(ns)
SF-36 MH	3.9 (0.4)	3.9 (0.5)	0.2	(ns)	3.2 (0.8)	3.7 (0.4)	−1.2	(ns)	3.5 (0.7)	3.7 (0.4)	−1.1	(ns)

*CBQ-oL* Colour Blindness Quality of Life scale, *SF-36 MH* mental health subscale of the MOS 36-Item Short Form Health Survey, *PMI* Positive Mindset Index

Comparisons between CB participants and NV controls are made using matched t-tests. For men there were 11 age-matched pairings from 16 CB participants and 18 NV controls, and for women there were 5 age-matched pairings from 5 CB participants and 20 NV controls. Comparisons between CB and NCV are made using matched t-tests. The 30 age-matched pairings were from 46 CB and 199 control participants

#### Factor structure of the CBQoL

After incomplete responses were eliminated, there were 91 (*N* = 69 CB and *N* = 22 borderline normal) participants in this analysis, including four participants who did not state their gender. This number exceeds the minimum of 85 participants required for factor analysis [14].

The principal components estimation resolved in nine iterations. The scree plot indicated that five factors were found. Together, these factors accounted for 76.58% of the variance in scoring after extraction. Two factors contributed less than 10% so were eliminated from further analysis in order to reduce the influence of relatively weak items. The remaining three factors accounted for 62.11% of the variance in scoring. The observed KMO of 0.911 indicated sound underlying factors. Bartlett’s Test of Sphericity was significant (*χ* ^*2*^ = 2527.577; *df* = 630; *p* = 1 × 10^−223^), indicating good factorability of the correlation matrix.

The above analyses resulted in the final version of the CBQoL questionnaire consisting of three subscales and 23 items (Table 2). The factor loadings are shown in Table 2. Cronbach’s *α* reliability for all 23 items was .979.

### Initial validation of the CBQoL

Table 3 shows that the CB participants scored significantly lower on the Ishihara and the three CBQoL subscales, but not significantly lower on the mental health subscale of the SF-36 HRQoL or PMI.

For the CB participants, there was a positive correlation between the Ishihara test and the CBQoL subscales: Health & Lifestyle: *r* = .533, *n* = 91, *p* = 5 × 10^−8^; Work: *r* = .599, *n* = 91, *p* = 5 × 10^−8^; Emotions: *r* = .315, *n* = 91, *p* = 0.002. This shows acceptable concurrent validity of the CBQoL for the Health & Lifestyle and Work subscales, though weaker evidence for the concurrent validity of the Emotions subscale. In contrast, the correlations between the Ishihara test and QoL subscales in the control group were weak (average *r* = .106).

In a further validation of the Health & Lifestyle and Emotions subscales, for the CB group only there was a statistically significant, moderate positive correlation between the CBQoL Health & Lifestyle subscale and the mental health subscale of the SF-36 (*r* = .297, *n* = 63, *p* = 0.018), and there was a statistically significant, moderate positive correlation between the CBQoL Emotions subscale and the PMI (*r* = .393, *n* = 63, *p* = 0.001). Although these correlations are weaker than ideal for concurrent validity, they are stronger than those for the control group (CBQoL Health & Lifestyle subscale and the SF-36: *r* = .200, *n* = 322, *p* = 0.0003; CBQoL Emotions subscale and the PMI; *r* = .057, *n* = 321, *p* = 0.308), which supports ‘known groups’ validity.

## Discussion

In this study we describe the development of a questionnaire measure of the QoL impact of being colour blind. The presence of CB was based on self-report, with confirmation by Ishihara testing. Both male and female CB participants scored significantly lower on the three CBQoL subscales than NV participants.

CB participants scored significantly lower on the Emotion CBQoL subscales than NV participants. This echoes the finding by Sullivan [4] that colour blind children may feel embarrassed about not knowing colours. The present study expands on the range of issues that may be seen in CB adults, including feeling anxious, depressed and lacking in self-esteem due to issues caused by being CB. CB participants did not score significantly differently to NV on the mental health subscale of the SF-36 HRQoL, nor on the PMI, though Table 3 shows that colour blind women had slightly more difficulty with SF-36 MH and PMI than their NV counterparts.

CB participants scored significantly lower on the CBQoL Health & Lifestyle subscale than NV participants. This echoes findings of Spalding [1] regarding problems seeing blood in stools, and [21] because we found that worse colour vision was correlated with the item (in Table 2) assessing confidence about taking coloured medication (*r*_*s*_ = .317, *p* = 3 × 10^−7^).

CB participants scored significantly lower on the CBQoL Work subscale than NV participants. This supports previous research finding that CB creates a barrier to entry to a range of occupations, and besides those occupations, problems are experienced with colours within many jobs that do not so obviously rely on colour [3].

These findings demonstrate that the negative effect of colour blindness on QoL can be profound. For example, a Cohen’s *d* of 0.8 or more is considered a large effect size, and the effect size of colour blindness on the Work subdomain of CBQoL is a Cohen’s *d* of 1.83 (the mean and SD data is in Table 3).

### Strengths of the study

In addition to its primary aim of developing the first QoL measure specific to CB, the present study provides a quantitative estimate of the impact of this common but often overlooked condition. This study is the first to assess the QoL impact of problems with colour vision in people with colour blindness compared to normally sighted controls. The three subscales show good psychometric properties in terms of factor loadings, Cronbach’s alpha and other relevant measures.

The CBQoL has further potentially useful applications e.g. in contrasting the effects of congenital vs acquired colour blindness, the effects of different types of congenital colour blindness (e.g. across protan, deutan, tritan axes) and the varying impact of different acquired diseases (e.g. retinal pathologies vs optic neuropathies). A future study of an intervention for people with acquired colour vision problems might use the CBQoL to measure changes in QoL from pre- to post-treatment.

### Limitations of the study

The cause of CB was not explored in this study. It may be that some forms of CB have a much less - or much greater - impact on QoL than we observed here. Future work will explore the variation in impact between congenital and acquired forms of CB.

The method of recruitment to this study may have led to sample bias. Most participants were recruited online, which limits the sample to those who had access to the internet and/or involved in colour blindness forums, or recruited through professional groups. This presumably contributed to a relatively high proportion of participants in each group being from a professional background, though this was not significantly different across the CB and NV groups. It is worth noting however that CB people may have career limitations and different employment due to colour difficulties than NV people [7]; inadvertent sampling bias towards professional groups may therefore exclude people whose careers have been most negatively affected by CB and therefore underestimate the impact of CB on work related QoL. Future research using the CBQoL might benefit from recruiting from non-online sources (e.g. in clinics) and recruiting more working class participants.

It is noted that the Ishihara test was not administered face-to-face by the research team but was administered online in uncontrolled conditions i.e. in participants’ own homes, where backlighting and monitor settings were not standardised by the experimenters; we cannot therefore externally validate the accuracy of the Ishihara scores. However the fact that the Ishihara scores were strongly correlated with the Work and Health & Lifestyle CBQoL subscales indicates some evidence of good concurrent validity.

## Conclusions

Colour blindness is a common condition, which can significantly impact quality of life. Problems may occur in health, lifestyle, emotions, and especially careers. Although congenital colour blindness occurs more frequently in men, colour blindness arising from a range of acquired conditions may affect both sexes. We suggest three things that might help these problems: firstly, incorporating CB screening into pre-school health checks; secondly, raising awareness of CB in the general population, and thirdly, where possible, workplaces should avoid colour coding (e.g. charts) where a non-colour alternative is possible. Practical approaches to the implementation of a CB-supportive working environment can be found at http://www.colourblindawareness.org/business/.

## Abbreviations

CB: Colour blind
CBQoL: Colour blind quality of life
CVD: Colour vision deficiency
HRQoL: Health-related quality of life
KMO: Kaiser-Meyer-Olkin
MH: Mental health
NV: Normal vision
PCA: Principal components analysis
PMI: Positive mindset index
QoL: Quality of life
SD: Standard deviation
SEC: Socioeconomic class
